# Dermoscopy and Histopathology of Darier Disease-Associated Nevoid Hyperkeratosis of Nipple and Areola

**DOI:** 10.5826/dpc.1103a44

**Published:** 2021-07-08

**Authors:** Siddhartha Dash, Biswanath Behera, Aparna Palit, Madhusmita Sethy

**Affiliations:** Department of Dermatology, and Venereology, and Pathology, All India Institute of Medical Sciences (AIIMS), Bhubaneswar, India

**Keywords:** Darier disease, Dermoscopy, Nevoid hyperkeratosis, Nipple

## Introduction

Nevoid hyperkeratosis of nipple and areola (NHNA) has been classified into 3 types: type 1 is associated with an epidermal nevus; type 2 with various dermatoses such as the Darier disease (DD), chronic eczema, and cutaneous T-cell lymphoma; type 3 is of unknown etiology. Despite the multifactorial association, a consistent morphological feature of NHNA is the presence of brown verrucous plaques involving the nipple and areola area, making the clinical distinction difficult [1].

## Case Presentation

**Figure 1:**
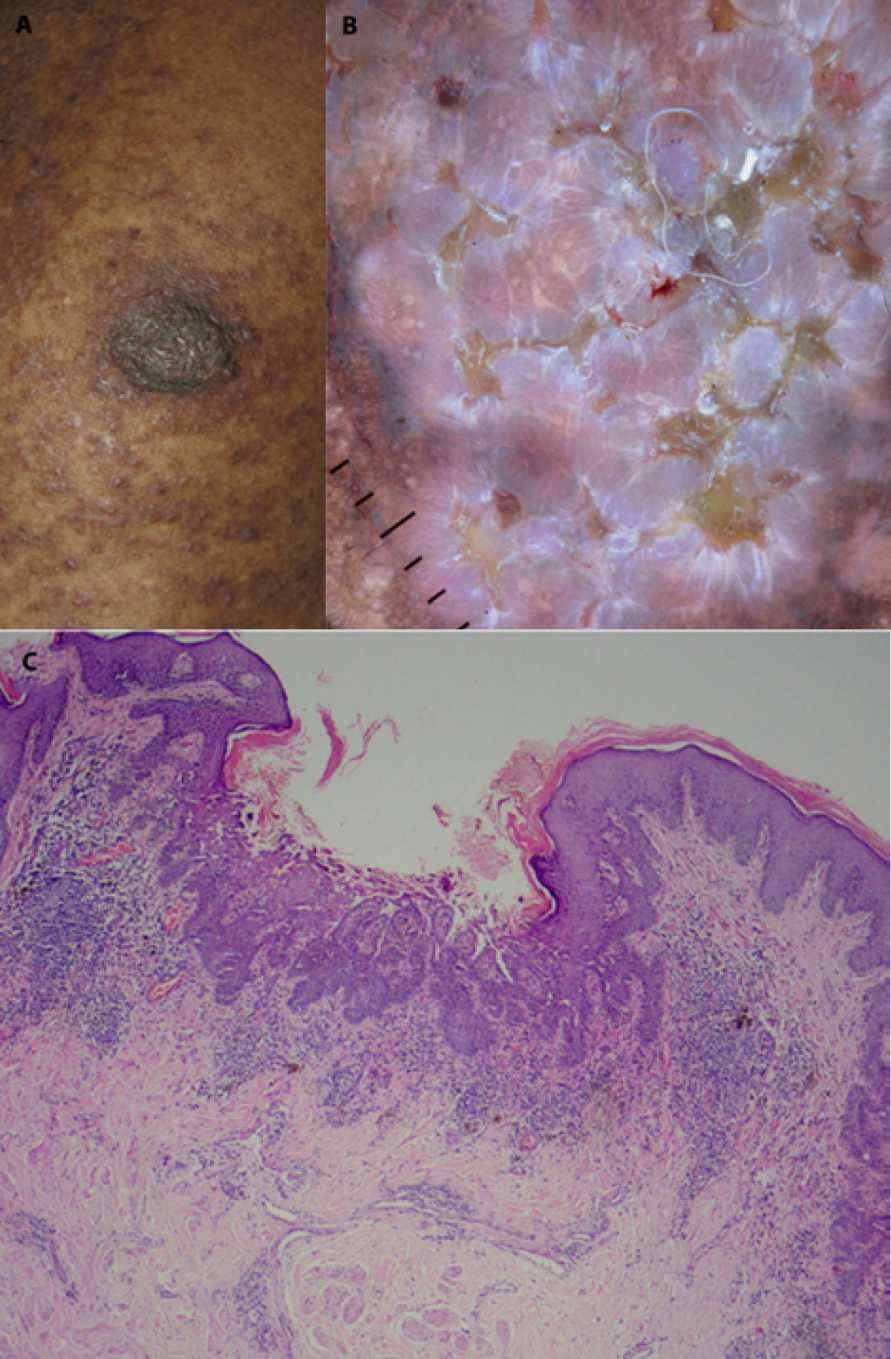
(A) Erythematous to gray-coloured verrucous plaque hindering the visibility of the nipple. (B) Dermoscopic examination showing varying shape central crater filled with yellow to yellowish-brown keratotic plug surrounded by white radial streaks, and outermost brown homogenous area and pigment network. The craters have an angulated border. (C) Histopathology from nipple-areola complex showing suprabasal acantholysis, along with corps ronds and grains at the base of a surface crater (H&E, ×50).

**Figure 2:**
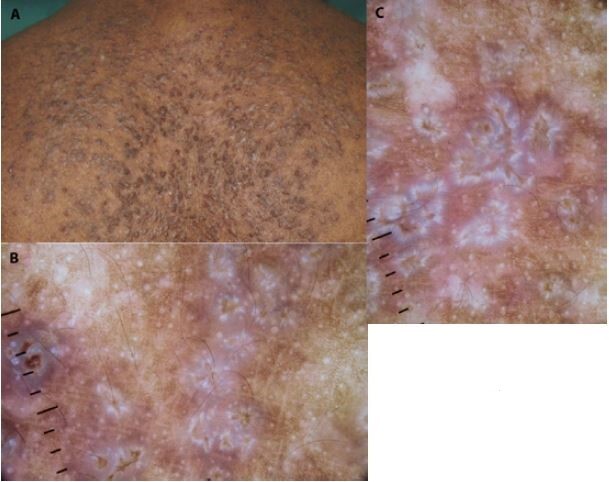
(A) Multiple erythematous to gray-brown verrucous papules over the back. (B) and (C) Early and advanced papules showing varying shaped central crater filled with yellow to yellowish-brown keratotic plug surrounded by white radial streaks, and outermost brown homogenous area and pigment network.

**Figure 3:**
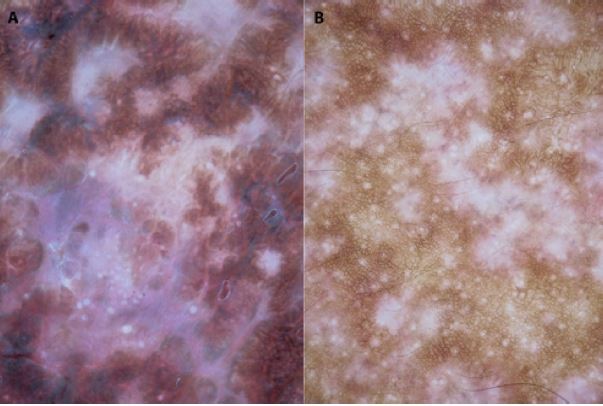
(A)Subsidence of dermoscopic features of nevoid hyperkeratosis of nipple and areola. (B) Improvement of the verrucous papules is evident by the disappearance of central crater, keratotic plugs, and white streaks.

A 25-year-old male with skin type V presented with multiple variably pigmented lesions all over the body since the age of 5. He reported a history of swelling affecting the bilateral nipple-areola complex (NAC) for 1 year. Lesions were foul-smelling and exacerbating during summer. He did not report a similar family history. On cutaneous examination, the bilateral NAC showed erythematous to gray verrucous plaques that hindered the visibility of the nipples (Figure 1A). Dermoscopic examination (Dermlite, DL4, 10× magnification) of the NAC revealed a central crater filled with a yellow to yellowish-brown keratotic plug surrounded by white radial streaks, and outermost brown homogenous area and pigment network (Figure 1B). The shape of the craters was variable with an angulated border. Histological examination of the plaque over the NAC, revealed a suprabasal acantholysis along with corp ronds and grains (Figure 1C). A diagnosis of nevoid hyperkeratosis of nipple and areola (NHNA) secondary to Darier disease (DD) was made. Besides, there were multiple erythematous to gray-brown verrucous papules scattered all over the body, more so in the seborrheic distribution (Figure 2A), which revealed a similar dermoscopic pattern irrespective of the size or duration of the lesions (Figure 2, B and C). The patient was treated with 30 mg isotretinoin capsules once a day. 2 months post-therapy, NHNA dermoscopic features and cutaneous lesions showed a significant improvement (Figure 3).

Dermoscopic features of NHNA are sparsely reported. Mazzella et al. reported multiple blue-gray globules and leaf-like areas in the case of type 3 NHNA [1]. In the index case, a similar dermoscopic pattern comprising of a central varying shaped crater surrounded by white radial streaks, and an outermost brown homogenous area to pigment network, was observed for NHNA and verrucous papules. This suggests the replication of DD dermoscopic features despite the varying morphological presentation, size, and location of the lesions. Lacarrubba et al. described a similar dermoscopic pattern for DD, consisting of polygonal, star-like, or roundish-oval-shaped yellowish/brownish areas of various sizes surrounded by a thin whitish halo, as reported by us [2]. In addition to differentiating other causes of NHNA, the observed dermoscopic pattern can be extremely helpful, especially for DD patients presenting with isolated hyperkeratosis of the nipple. In concurrence with clinical improvement, the disappearance of dermoscopic features, the central crater, and radial streaks, suggest the response to therapy.

## Conclusion

Here we reported a characteristic dermoscopic pattern found in a case of DD associated NHNA, which can be valuable for future diagnosis and for differentiating it from other NHNA causes.
